# A qualitative exploration of online forums to support resilience of rural young people in Australia

**DOI:** 10.3389/fpubh.2024.1335476

**Published:** 2024-05-22

**Authors:** Karen Carlisle, Peter Kamstra, Emily Carlisle, Anthony McCosker, Tracy De Cotta, Sue Kilpatrick, Artur Steiner, Bianca Kahl, Jane Farmer

**Affiliations:** ^1^College of Medicine and Dentistry, James Cook University, Douglas, QLD, Australia; ^2^University of Melbourne, Melbourne, VIC, Australia; ^3^Department of Media and Communications, Swinburne University of Technology, Melbourne, VIC, Australia; ^4^Social Innovation Research Unit, Swinburne University of Technology, Melbourne, VIC, Australia; ^5^University of Tasmania Launceston, TAS, Australia; ^6^Glasgow Caledonian University, Yunus Centre, Glasgow, United Kingdom; ^7^ReachOut Australia, Sydney, NSW, Australia

**Keywords:** rural, mental health interventions, online peer support forums, resilience, youth–young adults

## Abstract

**Introduction:**

Prevention and early intervention are crucial strategies for improving young people’s mental health and well-being. Building resilience is a key component of these strategies, especially among young individuals in rural areas who face well-documented mental health disparities. This study aimed to investigate how online mental health forums can contribute to enhancing individual resilience in young rural users.

**Methods:**

A sample of forum posts (*n* = 1,000) made by Australian rural users (18–25 years) on an online peer support mental health forum were qualitatively analyzed. The analysis was guided by themes derived from the literature on indicators of rural resilience.

**Results:**

Analysis of forum posts showed evidence of rural resilience in forum users. Online peer support forums offered a virtual space for individuals to establish social connections, experience a sense of belonging, share information, acquire knowledge, and offer mutual support. There were indications of increased self-efficacy among forum users, as they demonstrated their ability to implement strategies for better managing their mental health.

**Discussion:**

These findings significantly contribute to our understanding of how online forums can enhance resilience factors that are beneficial for young people living in rural communities. In the context of prevention and early intervention, this study illustrates the intricate connections between forum design and user activity with resilience outcomes, providing valuable insights into the underlying causal mechanisms. Consequently, it emphasizes the importance of incorporating such digital interventions as integral components of mental health service ecosystems.

## Introduction

This study explores how an online peer support mental health forum contributes to building resilience of young Australians aged 18–25 living in rural areas. Building resilience is an important protective factor as part of mental health prevention and early intervention strategies (1), and is particularly significant for the young, rural group we consider. Globally, mental ill-health is a major contributor to the burden of disease in young people. It is estimated that one in seven (14%) of those aged between 10 and 19 years experiences a mental health condition (2), with suicide being the fourth leading cause of death in 15–19 year-olds. In Australia, nearly two in five (39.6%) of those aged 16–24 years experienced a mental health condition in 2020–21 (3). Internationally, adolescence and young adulthood are high-risk life-stages when many common mental health conditions emerge, with approximately 75% of these conditions starting by the age of 24 (4, 5). Rural is the specific geographical focus here due to well-documented rural mental health deficits when compared with urban counterparts (6, 7).

Given the specific salience of adolescence as a time when well-designed interventions could influence life-long mental health and well-being, effective strategies to stop exacerbation of health issues are imperative. However, young people with mental health issues are less likely to access mental health services compared with the general population (8). Frequently cited barriers include perceived stigma and societal attitudes toward mental health, poor mental health literacy and prior negative experiences of mental health services as well as a preference for self-sufficiency (9, 10). With specific reference to young people experiencing mental illness in rural communities, the focus here, there is evidence of limited choice of, and access to, mental health services, cost associated with treatment, and cultural barriers (11, 12). Without adequate supports, rural young people can experience high rates of relapse, poor health outcomes, and reduced quality of life (11, 13).

Prevention and early intervention are key policy and practice-based strategies to enhance the mental health and well-being of young people. Implementation of these strategies aims to halt the onset or development of illness, thus minimizing impacts on individuals, families, and communities (1). Most mental health conditions arise from combined risk factors - biological, genetic, psychological, family-related, and social - which interact in complex ways. Protective factors, such as social connectedness, access to education and employment, and a stable family environment can reduce the likelihood of experiencing a mental health condition, enhancing a person’s mental health and wellbeing, or acting as a buffer against a person’s exposure to risk factors. This reduces the likelihood of becoming unwell (14).

By implementing preventive measures that reduce exposure to risks and increase exposure to protective factors, it is possible to reduce occurrence of mental health conditions across the community (1, 15). Early intervention aims to lessen ill-health duration and impact by identifying early signs of mental ill health (16). Effective prevention and early intervention can prevent the progression of mental health conditions and reduce the mortality and long-term morbidity often associated with these conditions, including premature death, social isolation, poor functioning, and reduced educational and vocational productivity (17).

This study considers how using an online mental health forum influences resilience. Building resilience has received increasing interest as foundational in informing design of preventative and early intervention, protective, approaches for mental health (14, 18). The term resilience is used across a variety of contexts, and sometimes in relation to structural features of communities, regions and their populations (19, 20). In this study, we focus on individual resilience. Individual resilience can be described as a composite psychological outcome generated through a process of building strength and coping strategies to deal with adversity and adapt to overcome trauma or other vulnerabilities (21). We define resilience using a framework of indicators of resources – social connection, learning, belonging, self-efficacy and adaptive capacity. These are acknowledged to assist in dealing with adversity in a rural context (21, 22). Rural people experiencing mental ill-health are understood to experience contextual layers of adversity including having to deal with isolation, service inaccessibility, hostile or declining environmental conditions and a set of social attitudes from living in close proximity with a limited number of people (7).

Evidence suggests that mental health interventions emphasizing protective factors that build resilience, can act as a psychosocial buffer for young people when exposed to stressors (23, 24). Research with young people living in rural Australia, for example, found that a school-based program which focused on active learning and peer interactions led to self-reported improvements in self-efficacy and application of healthy coping strategies when faced with adversity (25). There are also increased calls to include young people as active co-contributors in mental health interventions to build resilience by giving opportunities to express their ‘voice’ leading to a sense of ownership and agency for change in their own lives (17, 26, 27).

Given rising mental health need among young people and the potential of building resilience for prevention or early intervention, the question arises of how to enable equitable and ubiquitous access to services and supports. Young people might be considered “digital natives” who have grown up immersed in technology with access to the internet a normal part of everyday life. The Australian Bureau of Statistics, report on household use of technology highlighted that 98% of young people aged between 15 and 24 years used the internet with social networking and entertainment the most common activities this age group engaged in (28). This level of digital native-ness among young people means that online interventions should be increasingly regarded as a valuable aspect of mental health service delivery models for young people (29). Online delivery of mental health services has potential to address some of the workforce and mental health service maldistribution across rural areas (30) and to overcome some of the confidentiality and agency barriers rural young people experience when seeking help for mental health issues (31).

Within the available options for digital mental health services, online peer support forums have potential as a “virtual social space where people come together to get and give information or support, to learn, or to find company” p348 (32). Face-to-face peer support has been increasingly recognized as valuable in mental health, for both giver and recipient (33) and, more recently, this has also been shown valid where peer support is mutually given via online forums (34). Beneficial outcomes are likely due to the interesting phenomenon of value co-creation (35) in peer service delivery where services can be tailored by a service-giver to fill any ‘structural holes’ (36) experienced by a peer who asks for help and seeks that in language they can understand. Simultaneously, the giver of help experiences a sense that they are valued because their help is needed and appreciated by another. Mental health service providers often incorporate online peer-to-peer support forums as part of online and in-person interventions to enable people to connect, share experiences and provide social support and advice. Mental health online peer support forums used by health organizations are typically managed and moderated by trained staff and peer volunteers who are trained and have recognized experience and expertise (37). Studies exploring the experiences of young people using mental health online forums have reported benefits in terms of symptom alleviation, a reduction of feelings of isolation, feelings of belonging, information sharing and emotional support (38). This is, in part, due to online forums affording young people alternative opportunities to connect with their peers in a supportive environment to manage their mental health and wellbeing. What is less understood is how online forums as a prevention/early intervention strategy can contribute to enhancing protective resilience resources that are associated with mental ill health in young people, particularly for those young people living in rural areas.

This study adds important new knowledge, therefore, from qualitative analysis of a sample of 1,000 posts (2018–20) made by Australian rural users, aged 18–25 years, on the ReachOut online peer support mental health forum. The study specifically examined how the forum influences resilience of its young rural users. By exploring the interactions between online forum users, the study shows how resilience is built by exchanges that are facilitated and encouraged to address a specific context and that harness a set of practices that are clever and adaptive to the young person group in a changing milieu. By engaging the young people this helps to effect the ongoing freshness, vibrancy and relevance that engages users. Helping to build resilience is significant as part of mental ill-health prevention and early intervention strategy. Findings of this study contribute to understanding the role of specialist digital services as part of health systems for key demographics – here a young, rural group that is both at risk and hard-to-reach.

## Materials and methods

This descriptive qualitative study is part of a larger project called (Identifying and optimising the roles of online communities in building rural resilience), which aims to understand if, and to what extent, rural people experiencing mental ill-health can realize resilience through participation in online communities. In this study, we focus on exploring how using the ReachOut forum influences resilience-building among young people aged 18–25 and living in rural contexts.

### Study setting

ReachOut is Australia’s most accessed online mental health service for young people. It was established in 1998 and is run by a non-profit organization. It is national in scope and accessed by more than 2 million Australian users annually, with around 9% of these residing in rural areas (39). The online peer support mental health forum (henceforth “forum”) aims to provide a safe, inclusive, empowering space that improves mental health outcomes (40). Its’ objectives are to build young people’s awareness and capabilities to:

- Recognize indications that something is not right – before reaching a crisis stage;
- Understand the importance of accepting and working with their mental health and psychological distress issues;
- Engage in informal and formal help-seeking and self-management as needed; and
- Support social connectedness and promote a positive sense of self and wellbeing.

The forum is informed by a theory of change that depicts it as an intervention aiming to expand the protective factors for young people’s mental ill-health, leading to prevention or delay of the onset of mental health issues, reducing their incidence, severity, duration or frequency (39). The forum targets those with low to severe symptoms with whom ReachOut can intervene ‘early’ to facilitate access to more intensive support, while providing ‘adjunctive support’ (39). Adjunctive support refers to being used in conjunction with other services, potentially while on waiting lists or between appointments, but the forum is also suggested to act as an alternative support for young people who may have had negative experiences with mental health professionals (40).

Young people can access the forum via the ReachOut website. ReachOut is an ‘open access’ forum, meaning anyone under 25 with an internet connection can view and register to post on the forums. To make a post, registered users must select a pseudonym associated with their profile to ensure all posts remain anonymous. Young people use the forum voluntarily by choosing to post on existing threads, or by creating their own new thread. Existing threads include topics on managing mental health, self-care, relationships and other topics relevant to young people. During 2020, over 65,000 posts were made on the ReachOut forum (41). The forum has paid moderators who: establish a safe space for all young people, monitor adherence to community guidelines, and facilitate safe conversations about sensitive topics (e.g., suicide, self-harm, trauma). Moderators ensure users do not disclose personal information, remove spam content, and prevent prescriptive advice or abusive and triggering language from reaching the forums.

As well as these paid moderators with specific roles, ReachOut users can access additional capability-building and formal helping roles within the forum by volunteering to become ‘community builders’. In that role, they receive training and are encouraged to provide peer support, start threads and engage in online events – thereby helping to keep the online forum active, dynamic and vibrant through user activity. Active users who demonstrate natural leadership in their posting activity and are over 18 years old may be invited by ReachOut to become an unpaid ‘peer moderator’ after receiving additional training on how to effectively provide peer support beyond the entry level community builder program. Through these means, ReachOut builds confidence among users and a kind of ‘career development’ for them, while keeping the forum fresh, active and co-created.

### Data collection

To gain ethics approval to source ReachOut data, a data sharing agreement was developed between the research team and ReachOut that ensured sensitive forum data would not be shared with third parties. Once retrieved, the data was stored securely on university servers and remained unmodified other than being completely de-identified (42). The data sharing agreement was used to inform ethical approval for this study gained from Swinburne University Research Ethics Committee (R/2019/033).

In accordance with data governance practices, forum users consent to the use of their anonymized post data for research when they register to use the forum. In this study, to ensure data was de-identified, users’ online pseudonyms and other potentially identifying features (e.g., references to specific locations in posts) were removed prior to analysis. A sample of posts from 1 August 2018 to 31 December 2020 (inclusive) of de-identified and time-stamped forum posts was obtained (*n* = 80,174 posts) and cleaned for analysis. As this study focused on rural young people, posts made by rural-located users were selected-out by linking their post codes (provided when registering) to each of their posts and then selecting posts for Outer regional, Remote and Very Remote categories (i.e., not including posts for ‘Major cities’ and ‘Inner regional’ areas) as defined by the Australian Statistical Geography Standard (ASGS) Remoteness Structure (41). The ASGS categorizes locations based on population distribution and distance to services. To generate a manageable sample for labor-intensive qualitative thematic analysis, we selected a sample of 1,000 posts, including all Remote posts (*n* = 57), all Very remote posts (*n* = 23) and a random sample (using Excel feature) of 920 Outer regional posts.

### Data analysis

Forum post data were analyzed deductively for themes derived from the literature on indicators of rural psychological resilience (21, 22, 43–45). The process of theme development is described in more detail in the larger study (46). A deductive approach was used as we were interested in exploring the utility of applying established resilience themes from the literature to the data set. Table 1 shows the themes and the topics of posts coded to these. Regarding the focus of this study – i.e. that it involves rural-dwelling young people experiencing mental ill-health - we draw on ways resilience is construed through the sequence of studies undertaken by Berkes, Ross and colleagues cited above. In their studies, they combined a psychological perspective on resilience with a rural community development perspective (to acknowledge the nuanced challenges and thus strength and coping strategies required for dealing with generating psychological coping resources in rural communities). That is, resilience as broadly involving access to a set of resources: social support and the ability to access networks of contacts for support; learning or access to new knowledge and skills (43); a sense of belonging or inter-connection with a community or place (47); self-efficacy which relates to an individual’s belief about their capabilities to reach goals and to exercise influence over their lives (48); and adaptive capacity to enable adaptation and behavior change in relation to taking agency in changing circumstances (49). As we did more widely in our larger study (46), we applied this rural and psychological framing of resilience to analyze resources exchanged or developing through forum posting.

**Table 1:** Resilience themes and topics in data coded to themes.

Resilience themes	Description of what was coded to this theme
Social connection	Includes: (i) descriptions of relatedness or empathy between people. Captures examples of one person describing to another, their similar experiences; (ii) expressions of friendship and friendly encounters between peers. This includes people offering encouragement to one another, referring to each other as friends, thanking each other for trusted friendships and describing the value of the friendships.
Learning	Includes: (i) formal knowledge resources or where people *ask for* advice and information about practical things, e.g., how to access services. Also *sharing* online resources, strategies for coping with symptoms and best-practices for improved mental health; (ii) informal knowledge or where information or advice is shared that is about others’ lived experiences, e.g., asking or sharing how medications made them feel, how they navigate relationships and situations.
Sense of belonging (to the forum community)	Includes: (i) people initially joining and posting messages indicating they seek to belong to this community, e.g., introducing themselves, telling their story; (ii) posts that tell members they belong. This captures the variety of ways members try to make others feel included and valued. (iii) posts that reference the forum as a beneficial place. Includes naming the forum, discussing features of the forum like it’s a physical place and testimonials about benefits of the forum.
Self-efficacy	Includes where people describe (i) feeling they have lost control. We interpret this as meaning they are taking steps to regain control by being on the forum; (ii) where people post in such a way that they are asking for others on the forum to ‘hold them accountable’; (iii) where people tell their story as a kind of ‘unmasking’, ‘offloading’ or getting it out there, with sometimes explicit discussion and sometimes implied that this helps to move on.
Adaptive capacity	Captures where people describe how interacting on the forum has changed what they do, how they approach certain situations and how they have changed since interacting online. Any post that mentions a change in behavior, approaches to talking to doctors or any other relevant change in behavior is captured here.

Analysis of posts followed Braun and Clarke’s (50) thematic coding method. Four researchers (PK, JF, EC, and TD) initially read all of the posts in the sample, noting themes and other ideas independently. A codebook was developed outlining what would be included/excluded for each thematic code (See Table 1 for forum post topics coded to each theme). Using the codebook PK, EC, TD systematically coded forum posts independently using the qualitative coding software NVivo. This coded data was reviewed and discussed with the wider research team with agreement reached on inconsistencies. It was agreed that where consensus on coding could not be reached, that data was not coded. Following this stage, data was grouped by each thematic code and explored in-depth to understand how each post related to the resilience themes in Table 1. For this paper, the coded data was then reviewed and refined by KC by re-reading the posts/post extracts to ensure a close fit between the data and interpretation of them leading to their coding. This refined analysis was examined by PK and EC and any agreement reached about inconsistences. Some posts were coded to more than one theme.

## Results

### Evidence of resilience

**Figure 1:**
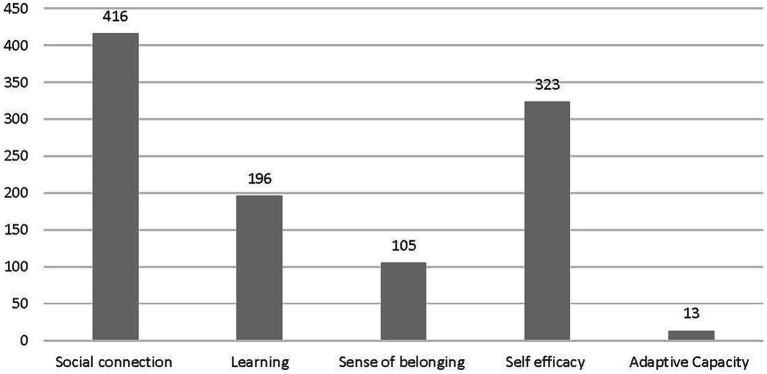
Number of posts coded to resilience theme (*n* = 1,000).

Below we show evidence that each of the resilience themes from the framework (see above Methods) was present in the forum data and how the themes were manifest in the data. Figure 1 provides an overview of the number of posts coded to the resilience themes.

### Social connection

The most frequently coded theme was social connection, with 416 posts coded to this topic. The resource of social connection was manifested in posts where users provided encouragement to other users, and shared experiences and expressions of friendship. Posts were mainly conversational in tone and included discussions about challenges with school, university and work, providing a context for users to connect. The following post illustrates how users shared thoughts about their everyday lives, in this case about challenges with their work environment.

*I feel imposter syndrome a LOT at work now! (It has a name!) I know it’s okay to make mistakes and that we learn from our mistakes, my current manager doesn’t have a relaxed attitude to mistakes as the previous one……* (Post #324)

Users connected through using words of encouragement and relating to each other about experiences they were going through together.

*I’m glad things are starting to feel a little better for you and hope they continue that way. How is the assignment coming along? How amazing is it going to be when we’re all finished for the year?! I know I cannot bloody wait* (Post #37)

Posts also reflected benefits of social connection particularly for people living rurally who may lack connection with others their own age in their local community. Being able to form connections online appeared to enable young people to know they are not alone in how they feel, as often the posts about social connection contained phrases like: *I can totally relate (*Post #226), and *you and I are going through some similar things* (Post #462).

Expressions of reassurance were shared through showing concern about others’ safety by reminding them they are connected to a wider, strong support network. Typical posts included ‘checking in’ and asking questions about the other person’s life or day.

*Hey I also wanted to check in and see how you’re doing? I’m hoping the absence means you’ve just been busy baking Christmas cookies and your rice bubble slices!! Sending you some positive vibes - you’ve got this. Don’t forget we are here okay? Ready to listen when you need someone! Thinking of you!* (Post #15)

How the forum operated also helped to facilitate social connections to happen. ‘Tag games’ was a tool used on the forum and refers to specific posts initiated by moderators aiming to acknowledge and encourage users who had contributed positively to the forum that week. Moderators would start tag games by posting questions for users to answer to encourage discussion and inspire new users to post as illustrated below:

*Awesome answer Since this is soo late (sorry guys!) I’m going to post today and tomorrow’s questions 4. Are comparisons bad and what can we do to help prevent upwards comparison? (i.e., thinking someone is prettier or better than oneself) 5. How can we promote positive thoughts surrounding body* (Post #236)

This type of post helps users to connect easily with one another - particularly new users; as it does not involve one to one chat. Once a user has answered a tag question, there is no onus to reciprocate. ‘Tag games’ enabled users who were seeking connection as their purpose is to bring about new connections between users with little proactive effort from users:

*… here’s our Friday Fives for creating this thread to talk about the recent attack in Melbourne and linking resources for help! for always being sympathetic and caring toward other users for opening up to friends about how they’ve been feeling….* (Post #884)

### Learning

Nearly a fifth of posts (*n* = 196) showed evidence of learning facilitated through gaining and taking onboard new information shared on the forum by other users. Some of this was based on users’ experiences, opinions or feelings and some was based on asking for or sharing sources of advice, knowledge or evidence. Users would access the forums to ask for advice on issues that were affecting their daily lives. Typically, those seeking advice would provide some contextual description about their circumstances to find people who had experienced similar issues.

*Because I am in a very small school, it is very difficult to find people with a similar mindset as me toward learning. What could I do to strengthen my mindset and lessen the chance of being negatively influenced in my situation? Much help would be appreciated (Post #14)*

Advice shared by users was often couched in similar experiences to the person posting or it would relate to the situation they were going through. At times young people in the forum would frame their advice with a question. This way of communicating the advice could be viewed as a less direct way of encouraging the person to consider the advice provided.

*Yeah I’m worried about that too!! Do you have the option to sit them on campus if you want to? I know with my uni you could register to sit them on campus and they have rooms set up with social distancing and stuff in place. I ended up applying for it cause I already don’t do well with exams so needed to keep things as normal as possible so am very thankful my uni has those measures in place! Could be worth checking out?* (Post #513).

*I think it’s important you try your best to work on supporting and relying on yourself for the next few weeks? Maybe chat with your psych and see what she suggests? (Post # 157).*

The forum was also used to exchange information about how to find professional help (as access to services is a common challenge in rural areas). Receiving lived experience advice from peers who have recently navigated the mental health system was often noted as helpful in supporting recovery, demonstrated here:

*Regarding the* [time I was with the] *crisis team, that is an interesting question. I am guessing perhaps about 7 months. However, I only stopped with them because the mental health nurse I had left and the new person I didn’t click with, and I was too scared to ask for someone different. Around the time I had started seeing a new GP who got my referral done for headspace and got me in quickly, so there really wasn’t much time where I was unsupported if that makes sense. Was there anything you wanted to ask about seeing a crisis team?*

*(Post #397)*

Users would ask for or receive practical tips for managing mental ill-health - for example, sharing ways to use crisis helplines. The following post is an example of advice on how to use helplines. It is interesting to note that the user has not written out the word “suicidal” in full as a tactic to avoid content being picked up and possibly removed by moderators.

Remember that you can always get immediate and temporary support from helplines and crisis lines if you are really struggling and are having s*cidal thoughts, it’s good to speak to someone… (Post #24)

Knowledge was sometimes obviously shared by moderators who would post and facilitate threads discussing practical ways to prevent mental ill-health, exemplified below:

*Welcome everyone Tonight our chat is all about ROUTINES! I’m facilitating the chat tonight with the lovely [name removed] & we have some builders who are going to help us out too* (*Post #*489)

### Belonging

Through connecting with one another on the forum, users began to form a sense of belonging to the online community and 105 posts were coded to this theme. Many posts were directed to other users being told they belong and were reassuring in nature.

*Sending you some positive vibes - you’ve got this. Don’t forget we are here okay? Ready to listen when you need someone! Thinking of you!* (Post #65)

Several posts depicted the forum as a space or place, drawing on features of the forum to help depict its place-ness. Posts were welcoming and reflected ‘safe space’ language.

*Hehe, don’t worry about the long posts, I tend to ramble on and on too! And I find that it’s super easy when you’re in a safe supportive place* (Post #698)

Users also described the benefits of belonging or being part of the forum in terms of finding support through difficult periods in their life.

*Thanks heaps I really do value everyone’s support on here because I’d have completely lost my mind by now otherwise!!* (Post #241)

Others discussed how joining the forum had furthered them in their journey of dealing with their mental health condition. At first, they appreciated receiving advice, and later, they experienced benefits from advising others.

*When I look back to when I first joined RO* [ReachOut]*, I would never have imagined I could be a mod* [moderator] *and be helping people the way I do now, but I did, and it started by helping give others advice when I can, and it also helped that I was taking suggestions and implementing them.* (Post #748)

Discussing the benefits of belonging, some users indicated a preference for using the forum when in need of immediate help rather than using crisis hot lines such as Lifeline (the most prominent Australian crisis help-line). Reasons for this were that they could connect with like-minded people and receive support from more than one person.

*I’m far too scared to make the call even when I really need it, and I’ve tried their online/text options but didn’t find them very helpful which is why I use ReachOut instead.* (Post #627)

*RO is my best source of support in terms of online/phone supports, I find everyone on here is just so amazing, understanding and accepting and it’s nice.* (Post #342).

*I think just knowing I can come on here and chat to like-minded people has been my favourite wellbeing activity this year. I am so glad I found this community because there is no other like it and for once I feel like i am supported and can speak out about how I’m truly feeling so thankyou to everyone on here!!* (Post #10)

### Self-efficacy

Almost a third of posts (n = 323) were coded to self-efficacy which can be understood as having skills and applying strategies that promote emotional coping. As an example, being able to identify things causing emotional distress and being able to make changes for the better or accept what cannot change, would be expressions coded to self-efficacy. Self-efficacy was evidenced in users discussing how they deal with their mental health in their day-to-day life and in forum threads such as one called ‘negatives and positives’, where users share positive changes they experience over time.

*I managed to walk to the end of my street AND I managed to go for a run on this quiet little track behind my house!! Double win!! Sorry, I know it’s pathetic but I’m on a bit of a high and just so proud of myself for actually doing it!! Feels amazing!!* (Post #682)

*I spoke to my psych today cause it’s just been too much for me and making me sick. She reminded me that I don’t have to put up with verbal abuse and be walked all over. She’s given me a couple of numbers I can call for actual legal advice for house shares so I might try calling them tomorrow to see what they say. I’m just trying not to think about it now and try chill out for the night* (Post #305)

Some posts illustrating self-efficacy responded to experiences shared on the forum and promoted a change in thinking or taking action.

*“Thanks for sharing your experience I really appreciate it! I have an appointment with my psych tomorrow and have just spent the last couple hours trying to word an email to her cause I’m not good at talking so hopefully she’ll bring it all up tomorrow and I’ll sort through some of it”* (Post #281)

Hey thanks! Things around that topic are doing just fine now, which is good, it feels I’ve had ten tone taken off my chest and I’ve already noticed a really positive difference in my general mood (Post #200)

Absence of self-efficacy was also evidenced and highlighted challenges faced by young people in rural areas when seeking help. Users expressed inability to get what they want because they are too young, cannot afford professional help, or legally cannot access a service due to their young age. Posts on this theme often implicitly noted lack of agency or were supportive responses to those experiencing the problem.

*I’m sorry to hear you felt that you weren’t able to answer the GPs questions honestly with your parents in the room.* (Post #365)

*I have severe insomnia and general anxiety. Paranoia decided to join, too. I’ve mentioned this to my family, and the first time, they were going to take me to a doctor. But they didn’t end up doing it. The second time I reminded them, they shook it off and said: “You’re fine, stop worrying”* (Post #11)

A prominent theme within those coded to self-efficacy involved discussions about managing interpersonal relationships, with n = 39 quotes on this issue. Some showed evidence of users helping each other to navigate complex interpersonal relationships in their offline life. The opportunity to discuss this with others online seems significant to prevention strategy as these relationship issues are depicted as deeply impacting users’ mental health. An example is given below where a user expresses problematical family conflict and they post repeatedly regarding this specific situation.

*There was another argument tonight, except this time it was my grandad being disrespectful and rude to my dad who was only trying to help. I don’t know why but it just makes me scared and feel really upset.* (Post #262)

### Adaptive capacity

Only 13 posts had evidence of adaptive capacity. Possibly this small number is because we only coded clear instances of behavior change to adaptive capacity. Some posts show evidence of people changing their behavior based on their forum activity. The following posts are examples of users describing how advice from other users led to changes in managing their mental health.

*I appreciate your, help, and so much, and I did talk to my boyfriend about it and I feel so much better.* (Post #18)

*Today I practiced self-care by reading back through my threads and taking note of all the wonderful suggestions and advice this community has shared with me so far.* (Post #229).

Users expressed capturing advice in the moment for later use - as reflected below - where the user states that seeing a post reminded them of the importance of self-care and prompted creating themselves a list of self-care methods.

*Thanks heaps for sharing this I came across it the other day when I was feeling really down and needing help and this was a great reminder of how important self-care actually is. I haven’t had much time to myself lately with uni exams and work but have finally got a day off today and have just put together a list that works for me in the hope I can just turn to it and pick something off the list to do when I’m feeling really low. Thanks for the little reminder!* (Post #645)

## Discussion

Supports that prevent or delay onset of mental ill-health by intervening early are critical for rural young people who are a particularly high-risk group (17). Building and enhancing protective factors – understood as supported, here, through resilience-building– is central to prevention and early intervention strategy. In this study, through qualitative analysis of 1,000 posts, we found that key resilience resources are built through young peoples’ engagement with the ReachOut forum, a service intentionally designed and targeted at users aged 18–25 years. The study found that through fostering young people’s exchanges of messages on the forum, a safe and trusted space for social connection, opportunities for learning and the building of self-efficacy, is created.

The forum supports young people in rural communities by helping them to reach specific resources that are hard to attain in rural places due to stigma, lack of privacy and lack of access to services and amenity. Finding others who are like you can be a challenge in rural places (51), so forums help young people to gain access to empathetic peers. Prescott et al. also described young peoples’ experiences of online forums as supportive environments to access information which may not be readily available offline (52) Specifically, young people can gain access to resources that are key to wellbeing, fulfilment and capability. Social relationships and a sense of belonging are important among these as determinants of mental health and predictors of quality of life and health outcomes (53). Social connection was enacted on the forums in several ways, by users, through encouragement, expressions of friendship (checking in) and sharing of experiences. Paid forum moderators and volunteer peer moderators, who are hidden but substantial co-creators of forum activity, facilitate connections through applying intentional tactics and techniques such as ‘tag games’ to engage users with each other. Moderators also curate the forum as a space of safety by carefully managing content to remove negative or triggering posts, while encouraging posting and development of forum users’ conversations. Peers in ‘community-builder’ roles keep forums active by posting and making encouraging responses to others. This seamless co-creation of a safe space fostered a sense of belonging and mutual support as a forum community which was expressed as beneficial for users particularly when faced with difficult situations, such as negative experiences in their community life. While the forum was not designed to manage crisis situations, some users would seek immediate help online. Reasons for this help seeking behavior are linked to understanding forum users as people who have empathy from sharing a similar situation (54), and from repeated, safe interactions making the forum a trusted support network (55). This might be contrasted with negative experiences of other ‘on-premises’ mental health services that young people might have used (40).

Young people engaged with the forum to learn from others and share their knowledge in return – particularly around seeking advice about preventing and managing mental health as well as navigating the mental health system. Use of forums for practical advice has been previously highlighted by other studies (38, 56). Of interest here is the experiential knowledge exchange and the value ascribed to the forum as a resource for sharing individual’s lived experiences. Many posts were grounded in personal experiences of managing users own mental health and contrasting difficult experiences within the public mental health system. This suggests forums accrue a form of legitimacy as an authoritative space to offer credible strategies to others and to seek advice from peers who understand you (37). This quality of forums is particularly relevant for the forum users who are living in rural, often isolated communities with limited access to appropriate mental health services. The availability of a 24/7 anonymous online forum is a valuable resource for young people distant from other services and perhaps isolated, lacking access to transport and agency, in rural places.

Another aspect of resilience which was prominent in the forum was evidence of self-efficacy. Self-efficacy can influence individuals’ thinking, feelings and behavior and is a strong predictor of health behavior change (57). Self-efficacy features in key program objectives of ReachOut online communities around accepting and working with mental health issues and engaging in self-management (39). Self-efficacy was manifest where forum users commented about taking control or working on changing their circumstances. This was often catalyzed by connecting with others with comparable experiences. This study provides insights into the role of connecting with others in similar circumstances and sharing knowledge via online forums as a mechanism influencing self-efficacy. Other studies have emphasized this contribution, showing that people sometimes use forums to record their feelings and observations, and to gain support during times when they are facing challenging circumstances (58).

**Figure 2:**
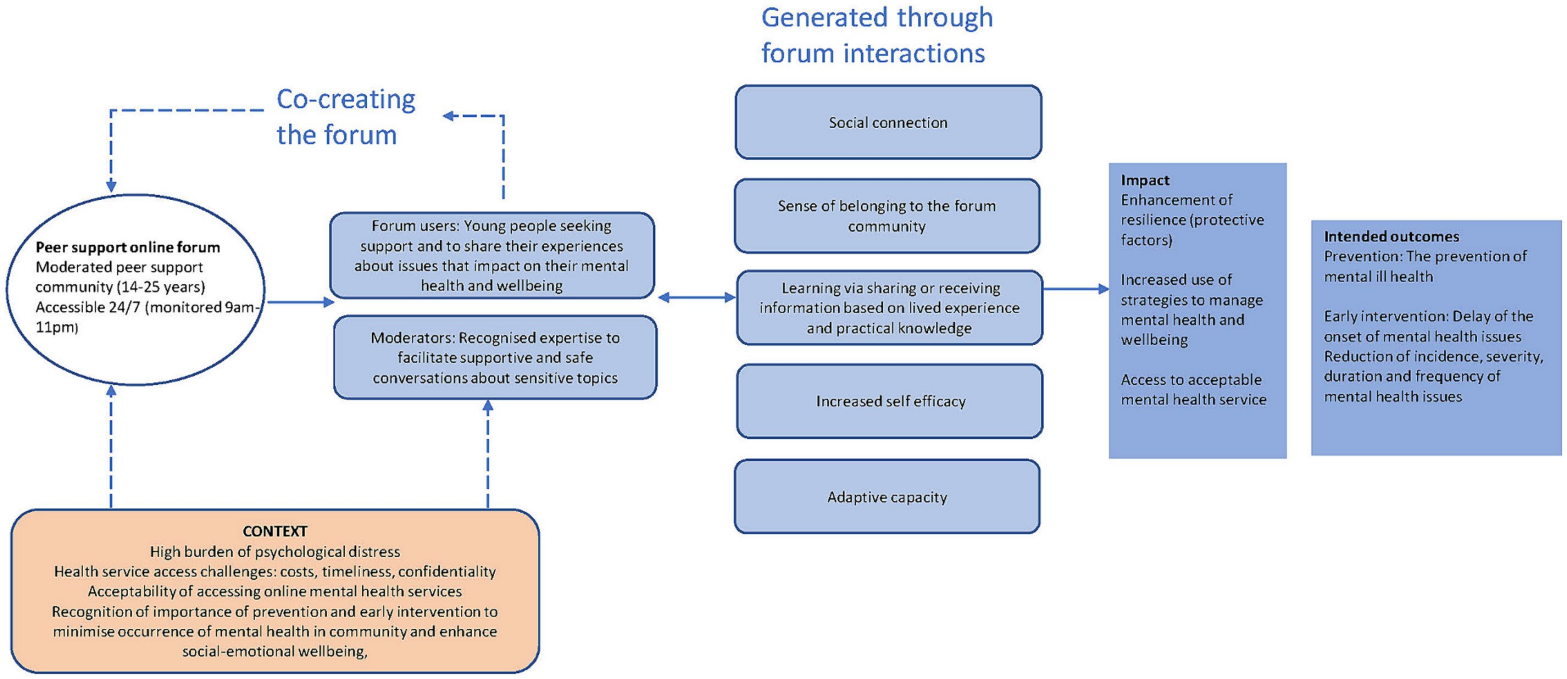
How the ReachOut online peer support forum works as a prevention and early intervention for rural young people.

The intentional design of the forum is significant to how forums contribute as a prevention and early intervention support. Figure 2 depicts how the forum is designed to address specific contextual conditions experienced by young people. A range of tactics are used (e.g., intentionally applied ‘games’, encouragement of users through different roles they may be assigned, removing sensitive and triggering posts) to enable co-creation between forum users (who exchange emotional attachment, support and information), and moderators. The co-creative activity creates a space where it is the norm to exchange knowledge and information in friendly, supportive ways, meaning people make positive connections with each other and build a reciprocal community. The focus of ReachOut is increasing self-efficacy so moderators are able to influence the flow of activity and information as positive, enabling and exchanging coping strategies. These, in turn, lead to instances of changed behavior and attitude, understood by us here as bringing the resilience resource – adaptive capacity. The impact of co-creating these resilience resources is raising the level of social factors protective to wellbeing (connection and belonging). The forum helps young people to manage their mental health and minimize symptoms by gaining strategies tailored to their age group and circumstances as provided by and discussed by, their peers. In addition, young people living in rural communities are afforded access to a mental health service they experience as acceptable – that is, which is available when needed, and addressing issues such as confidentiality and stigma. This acceptable online service is, unfortunately, often to be contrasted with hard to access ‘on-premises’ services that may be inappropriately designed for young people (59). Based on existing evidence of what contributes to prevention and early intervention, by helping to build resilience, and enable protection and service access, we can say that the ReachOut forum conforms to depictions of a prevention and early intervention service or intervention. That is, is likely to help in delaying the onset of mental ill health and reducing mental ill-health duration and severity.

This analysis suggests that online peer support mental health forums have a role to play as part of the suite of mental health interventions offered for young people in rural communities. With an ever-increasing demand for mental health services, service providers should find online forums fruitful to complement other modes of service delivery to facilitate timely access to help. Forums might be understood as particularly useful for young people as they tend to lack power and agency (in rural places, this includes potentially exacerbated lack of access to services through lack of agency around transport) (60). They represent the potential of a private space, easily accessible, where young people can go to be themselves, express themselves and explore identities that are under-represented in their immediate locale. Forums are spaces where parents, teachers and ‘grown-ups’ cannot prevent their expression or monitor them – providing, of course, they have internet access, which is a question in itself because rural places still experience lower digital inclusion compared with other demographic groups.

### Strengths and limitations

A strength of the study is the data collection method adopted. The data set that we analyzed comprised of 1,000 forum posts made directly by young people from outer regional, rural and remote Australia providing a rich, authentic source of information about questions and challenges, and tactics in addressing them, for this hard-to-reach population. Sourcing and analyzing data ‘already there’ helps circumvent the need to create and obtain data using surveys, interventions or other requests for data collection. Findings may be limited due to the deductive application of the resilience framework to forum posts, however the framework is based on a chronology of work specifically about rural psychological resilience, providing a framing specifically tailored to context. Other resilience frameworks and other methods of surfacing protective factors, from the data, might have been used in analyzing the data. We mentioned there are different ‘levels’ of users and indeed moderators whose posts may be in the dataset. It is impossible to separate out these types of actors within the dataset so we cannot understand any distinct differences between user status, the nature of posts and how dynamics within the forum might affect what is posted and significantly, what is not posted (61).

### Implications

This study provides evidence of links between activity on the ReachOut forum and building of resilience factors that are beneficial to young people as part of prevention and early intervention supports and services. It is well documented in the literature that the use of online mental health interventions for young people can increase access to mental health services that are timely, acceptable and can improve mental health outcomes for users (37, 38, 56, 62, 63). This forum and its building of resilience can be viewed as additionally significant to rural young people as they navigate a distinct context and set of additional challenges in addressing their mental ill-health. While there have been other studies highlighting the benefits of online mental health forums, this study explicitly links forum activity with resilience outcomes and provides fine-grained evidence that informs depiction of causal mechanisms (as illustrated in Figure 2).

This study also examines the capabilities of ReachOut as a forum operator in crafting and curating the desired outcomes to happen - particularly in addressing its goal of generating self-efficacy. It cleverly does this through combinations of skilled staff managing the forums, moderators, experienced users (peer moderators and community builders), teaching and skilling peers, moderation practices, games and tactics. Further, this practice does not seem codified and is adaptive and represents an example of benefits or good outcomes from more adaptive and co-creative practices in health and wellbeing services.

Finally, the study highlights the significance of this type of digital intervention – that enables young people to escape the surveillance of life in physical communities, is open and inclusive, available at all times and confidential. As we have noted, a beneficial set of characteristics of a service for rural young people who live in highly socially monitored contexts of few services, embedded in communities where everyone knows everyone else. While ReachOut understands this service as adjunctive and complementary, our findings suggest it is perhaps *an essential service*. If we are to understand young people as digital natives, the study highlights that service systems – and perhaps particularly rural service systems - should now consider online options as a routine part of adequate service ecosystems.

## Data availability statement

The data analyzed in this study is subject to the following licenses/restrictions: The datasets generated for this article are not readily available because signed data sharing agreements by partner organizations and Swinburne University used to receive ethics approval prevents us from making sensitive mental health data available. Access to the de-identified data is considered on request by the participating organization. Requests to access these datasets should be directed to karen.carlisle@jcu.edu.au.

## Ethics statement

The studies involving humans were approved by Swinburne University Research Ethics Committee. The studies were conducted in accordance with the local legislation and institutional requirements. The ethics committee/institutional review board waived the requirement of written informed consent for participation from the participants or the participants’ legal guardians/next of kin because in Australia people who sign up to use ReachOut forums, agree that their de-identified posts can be reused for research.

## Author contributions

KC: Conceptualization, Formal analysis, Investigation, Methodology, Writing – original draft, Writing – review & editing, Funding acquisition. PK: Conceptualization, Data curation, Formal analysis, Investigation, Methodology, Project administration, Writing – original draft, Writing – review & editing. EC: Conceptualization, Formal analysis, Funding acquisition, Investigation, Methodology, Writing – original draft, Writing – review & editing. AM: Conceptualization, Formal analysis, Funding acquisition, Methodology, Writing – review & editing. TC: Formal analysis, Methodology, Writing – review & editing. SK: Conceptualization, Formal analysis, Funding acquisition, Methodology, Writing – review & editing. AS: Conceptualization, Formal analysis, Funding acquisition, Investigation, Methodology, Writing – review & editing. BK: Formal analysis, Writing – review & editing. JF: Conceptualization, Formal analysis, Funding acquisition, Methodology, Writing – original draft, Writing – review & editing.
